# Dissecting the Role of the Extracellular Matrix in Heart Disease: Lessons from the *Drosophila* Genetic Model

**DOI:** 10.3390/vetsci4020024

**Published:** 2017-04-24

**Authors:** Chris J. R. Hughes, J. Roger Jacobs

**Affiliations:** Department of Biology, McMaster University, 1280 Main St. W., Hamilton, ON L8S 4K1, Canada; hughec7@mcmaster.ca

**Keywords:** cardiomyopathy, ECM, remodelling, Integrin, Collagen, MMP, TIMP, *Drosophila*, model organism, genetics

## Abstract

The extracellular matrix (ECM) is a dynamic scaffold within organs and tissues that enables cell morphogenesis and provides structural support. Changes in the composition and organisation of the cardiac ECM are required for normal development. Congenital and age-related cardiac diseases can arise from mis-regulation of structural ECM proteins (Collagen, Laminin) or their receptors (Integrin). Key regulators of ECM turnover include matrix metalloproteinases (MMPs) and their inhibitors, tissue inhibitors of matrix metalloproteinases (TIMPs). MMP expression is increased in mice, pigs, and dogs with cardiomyopathy. The complexity and longevity of vertebrate animals makes a short-lived, genetically tractable model organism, such as *Drosophila melanogaster*, an attractive candidate for study. We survey ECM macromolecules and their role in heart development and growth, which are conserved between *Drosophila* and vertebrates, with focus upon the consequences of altered expression or distribution. The *Drosophila* heart resembles that of vertebrates during early development, and is amenable to in vivo analysis. Experimental manipulation of gene function in a tissue- or temporally-regulated manner can reveal the function of adhesion or ECM genes in the heart. Perturbation of the function of ECM proteins, or of the MMPs that facilitate ECM remodelling, induces cardiomyopathies in *Drosophila*, including cardiodilation, arrhythmia, and cardia bifida, that provide mechanistic insight into cardiac disease in mammals.

## 1. Introduction

The heart is a dynamic organ, modifying its morphology and molecular structure in response to physiological changes through embryonic organogenesis, growth, exercise, and aging. Much of what is known of the genetic basis of heart formation and function has emerged from genetic models of heart function such as the mouse and zebrafish. There is but a single invertebrate genetic model for heart development and aging; the fruit fly *Drosophila*. Upon first glance, the fruit fly would seem an unlikely candidate for cardiac research; its heart is a dorsally-positioned, linear tube that is not essential for the delivery of oxygen to tissues and has no stem cells [1,2,3]. Therefore, why then have dozens of laboratories published over 1500 papers on this arcane model? The answer lies in the remarkable conservation of gene identity, expression and function between the fly heart and the hearts of mammals of medical or veterinary significance. Insights into gene function in the fly translate rapidly to vertebrates. Here we shall briefly review the fly model and the extent of cardiac gene conservation, with focus on one family of genes required to establish and maintain the extra cellular matrix (ECM); the matrix metalloproteinases (MMPs). Genes of the ECM underlie morphological adaptations in the heart that are linked to numerous cardiac diseases present in mice, dogs, pigs, and humans [4,5,6,7,8]. In this review, we shall illustrate how insights into gene function from the *Drosophila* model are relevant to our understanding of the basis of mammalian disorders.

## 2. ECM Regulation and Cardiac Dysfunction

The ECM is a dynamic three-dimensional network of proteoglycans, glycoproteins, and fibrous proteins linking and protecting the intercellular regions within organs and tissues, including the heart (reviewed in [9,10]). It serves as a scaffold, providing structural support to organise cells, transmit tension through tissues, and mitigate damage from mechanical stressors [11]. ECM remodelling is key to cardiac dysfunction and repair; the disruption of structural and regulatory ECM proteins is linked to the progression of myriad heart and vascular diseases. Increased ECM deposition is a hallmark of dilated cardiomyopathy (DCM), hypertrophy, and heart failure in humans (reviewed in [12,13,14,15]).

Congenital, pathological, and age-related cardiac disorders are a leading cause of death amongst many mammals, including humans and canines [16,17,18,19]. Heart diseases affecting the ECM, such as congestive heart failure and cardiomyopathy, are common to many dog breeds [20]. Amongst smaller dogs, mitral valve disease accounts for the highest number of cases of non-congenital cardiac failure, whereas cardiomyopathy is prevalent amongst larger dogs [21,22]. DCM has been characterised in Doberman Pinschers [23,24], Great Danes [25], Boxers [26,27], and Irish Wolfhounds [28], while sub-aortic stenosis has been examined in Newfoundlands [29,30] and Golden Retrievers [31], and ventricular arrhythmia is found in many breeds (reviewed in [20,21]).

Many cardiac disorders are difficult to elucidate due to the existence of complex gene and protein interactions, and functional redundancy. These disorders might be examined within simpler systems more amenable to manipulation, such as the fruit fly *Drosophila melanogaster*. Owing to the functional and compositional similarities between fly and vertebrate hearts, the study of cardiac disease gene candidates in *Drosophila* has the potential to illuminate genetic interactions underlying polygenic syndromes, which may not be readily determined in other organisms given the limitations imposed by life history traits and the availability of genetic tools (reviewed in [32,33,34]).

## 3. The *Drosophila* Model

*Drosophila* is a proven and powerful model for the study of cardiogenesis, cardiac aging, and heart disease [33,35]. This system boasts numerous advantages over vertebrate models owing to its relative simplicity, short generation time, and the availability of genetic tools. The diversity of genetic approaches is reviewed more comprehensively in [36]. Moreover, a sizeable library of mutant and transgenic strains is available through various stock centres [37,38,39].

### 3.1. Simplicity and Homology

Compared to vertebrates, *Drosophila* possesses a smaller genome encoding fewer protein variants (reviewed in [40,41,42]); *Drosophila* has fewer than 15,000 predicted protein-coding genes [43,44] compared to the (approximately) 19,000 in humans and mice [45,46]. As a result, a genetic approach may be utilised to manipulate the expression or structure of entire gene families simply by generating mutant alleles or inserting transgenes. This has enabled the inducible switching of, for example, all Integrin function or all endocytosis within intact organisms [47,48]. Furthermore, the expression of mutant isoforms of human genes in *Drosophila* can recapitulate human cardiac disease [49,50].

The *Drosophil*a heart, or dorsal vessel, is a linear valved tube, and thus structurally simpler than the looped and multi-chambered vertebrate heart. Both hearts are derived from developmentally homologous lateral mesodermal precursors and share the same pattern of specification and medial migration to the midline to form a cardiac tube (reviewed in [32]). Cardiogenesis in vertebrates and invertebrates involves many conserved molecular mechanisms [51,52]. For example, the transcription factors and signalling cascade specifying cardiogenesis are highly conserved; homeobox transcription factors activate a transcriptional cascade in the pre-cardial mesoderm that promotes cardioblast (CB) specification and later differentiation [53,54]. The homeobox transcription factor Tinman (Tin), which specifies the visceral and cardiac mesoderm, is homologous with mammalian Nkx2-5, though these differ in their deployment and may have adapted unique cardiogenic roles [55,56]. Tin is regulated by the TGF-β growth factor Decapentaplegic (Dpp), a BMP2/4-homologue [57,58]. Pannier, involved in CB specification in *Drosophila*, is homologous with GATA4 [59]. *Drosophila* possesses a single Mef2 gene, Dmef2, which is expressed in myocardial precursors [60,61]. Its *Hand* gene, encoding a basic helix-loop-helix (bHLH) protein expressed in cardiac precursors, has orthologues in vertebrates, including chicks and mice [51,62,63,64]. Vertebrate COUP-TF/NR2F is homologous with *Drosophila*’s Seven-up (Svp) [65], and Islet-1 (Isl1), which is involved in cardiac precursor specification, has a *Drosophila* counterpart in Tailup (Tup) [66].

Proteomic analysis reveals a general conservation of function between *Drosophila* and vertebrate cardiac proteins, which retain critical domain and structural similarities despite lower levels of over-all sequence identity [67]. This includes significant conservation of structural ECM proteins and receptors between *Drosophila* and mammalian species; for instance, Collagens, Laminins, Perlecan, and Integrins are all present in *Drosophila* (reviewed in [67]). Conserved proteins include those mapped to known vertebrate mutations resulting in cardiac disease and dysfunction [68]. For example, proteomics has identified Troponin-T as a conserved protein, which can trigger cardiomyopathy in humans if mutated in the Tropomyosin binding region. Similar mutations in the *Drosophila* homologue causes restrictive cardiomyopathy [69].

### 3.2. Life History

Unlike mammals, which may survive for years or decades and may require months or years to become reproductively competent, *Drosophila* species are short-lived, with an average lifespan of less than 100 days under normal laboratory conditions, and a generation time of only ten days. This facilitates longevity studies examining the effects of heart-related genes on aging [1,70,71]. Although heart function is necessary for adult survivorship, a poorly functioning heart is sufficient throughout embryonic and early larval stages of *Drosophila* growth, since the oxygenation of tissues occurs through the tracheal system (reviewed in [72]). This allows for the study of severe mutant or mis-expression phenotypes that, in other organisms, would prove lethal in early development.

### 3.3. Generating Genetic Mosaics

Although CRISPR-mediated approaches to genetic modification are becoming increasingly prevalent (e.g., [73,74]), the historical advantage of *Drosophila* over other genetic models lies in the ability to insert novel genetic sequences (such as gene enhancers, inverted sequences for RNAi, exogenous genes, or fluorescently tagged sequences), or to interrupt endogenous genes, using modified mobile genetic elements (e.g., P-element transposons) [75,76,77,78]. Temporal or spatial genetic mosaics can be created through site-directed DNA recombination with transposons containing yeast recombinase [79,80,81]. Mitotic recombination can be induced by tissue-specific enhancers, temperature shifts, or drugs to generate gain-of-function mutants or loss-of-function/genetic null mutants [79].

The Gal4/UAS system, originally adapted from the yeast *Saccharomyces cerevisiae*, has found common usage in *Drosophila*, enabling the precise spatial regulation of specific gene transcription [82,83]. This system can be exploited to drive the expression of transgenes (such as fluorescent reporters), or induce mis-expression, either through over-expression or RNA interference (RNAi)-mediated knock-down [84]. Temporal control can be exerted with the expression of a temperature sensitive inhibitor of Gal4 [85].

### 3.4. In Vivo Imaging

A key advantage of *Drosophila* in the study of the heart is the ease with which live imaging can be performed. Transgenic *Drosophila* can express fluorescent reporters in vivo by way of enhancer or protein traps. Enhancer traps are fluorescent reporter proteins (e.g., green or red fluorescent protein; GFP/RFP) expressed under the control of the endogenous enhancer [86,87]. Protein traps result from the insertion of a fluorescent gene transposon into a native gene, adding the GFP/RFP coding sequence as a new exon within the functioning endogenous protein [86]. Fosmid constructs may be used to insert transgenes encoding C-terminally tagged proteins [88]. The levels and localisation of these proteins can then be assessed within a living system [89,90,91].

The *Drosophila* embryonic and larval cuticle is translucent, and many internal structures remain visible in the live organism. Cardiogenesis may be observed in embryos using fluorescence microscopy [1,89]. The internal structures of the larger and more opaque larvae may be imaged using optical coherence tomography (OCT) [49]. OCT is non-invasive and high-speed, providing a rapid, high-throughput means of quantifying or qualifying contractility, rhythmicity, and heart chamber size and topography [1,92,93].

## 4. The *Drosophila* Heart

### 4.1. Early Morphogenesis

**Figure 1:**
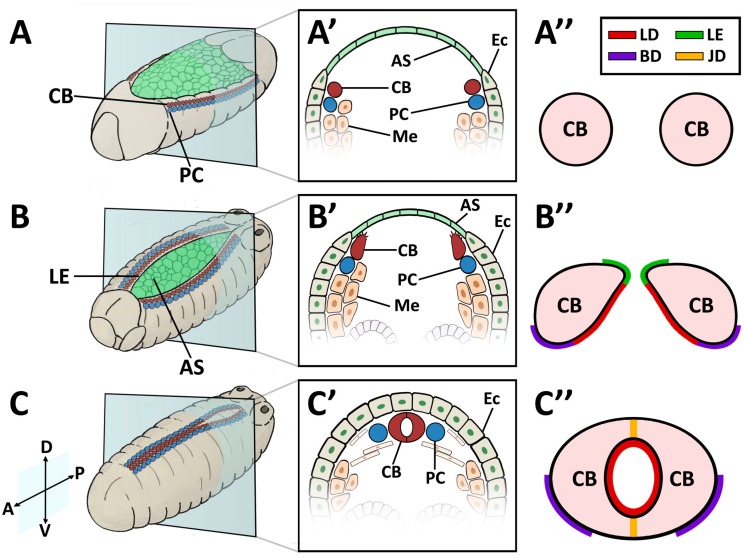
Cardioblast (CB) migration during *Drosophila* embryogenesis. (**A–A”**) Stage 12–13 embryo. (**A**) Perspective view of a whole embryo showing cardiac precursor cells associated with the dorsal ectoderm prior to dorsal closure; (**A’**) Cross-sectional impression showing the lateral-most mesodermal cells, which have become specified as CB precursors; (**A”**) CB precursors prior to the extension of the motile apical domain. (**B–B”**) Stage 14–15 embryo. (**B**) Lateral ectoderm and CBs migrate dorso-medially and displace the amnioserosa; (**B’**) Cross-sectional impression showing Leading Edge activity. CBs extend a motile apical domain towards the midline; (**B”**) CBs assume a teardrop shape with the Cadherin-rich apical domain forming a Leading Edge. The basal domain is quiescent. The pre-luminal domain localises Dystroglycan, βPS-Integrin, Multiplexin, and the Slit-Robo complex. (**C–C”**) Stage 17 embryo. (**C**) CBs make contralateral contact at the midline upon dorsal closure. (**C’**) Cross-sectional impression showing paired CBs at the dorsal midline, flanked by PCs; (**C”**) Formation of the heart tube. CBs form a dorsal and ventral seam at the Cadherin-based junctional domain to enclose a lumen. Dorsal at top. A: anterior; AS: amnioserosa; BD: basal domain; CB: cardioblast; D: dorsal; Ec: ectoderm; JD: junctional domain; LE: Leading Edge; LD: luminal domain; Me: mesoderm; P: posterior; PC: pericardial cell; V: ventral.

In contrast to vertebrates, *Drosophila* possesses an open circulatory system of low hydrostatic pressure. The insect circulatory system contains hemolymph, a nutrient- and hemocyte-rich interstitial fluid that directly bathes tissues and organs, but which is not essential for oxygen transport (reviewed in [94]). The dorsal vessel is a linear tube formed during embryogenesis. *Drosophila* cardiac precursors arise in a manner analogous to their vertebrate counterparts [95,96]. As the mesodermal precursors enter through the ventral gastrulation furrow, they flatten to associate with the interior (basal) side of the ectoderm. The lateral-most mesodermal cells receive the strongest ectodermal signal from the Dpp (BMP) and Wingless (Wnt) ligands, whose activities converge to activate heart-specific transcription factors, beginning with Tin (Nkx2-5) (Figure 1A). Subsequently, the lateral ectoderm migrates to the dorsal surface of the embryo to displace the amnioserosa, a transient tissue overlaying the yolk (Figure 1B). Tin-expressing heart precursors maintain an association with the dorsal ectoderm and accompany its migration. As the ectodermal cells approach their contralateral partners upon dorsal closure, the post-mitotic heart cells (CBs) migrate independently to meet their partners also [89,97]. The CBs assume a characteristic teardrop shape, whereupon the ectodermally-exposed edge of the apical surface extends motile processes medially, while the interior edge of the apical surface remains quiescent (Figure 1B’). This motile domain forms a Leading Edge, and exhibits guidance behaviour typical of Collective Cell Migration [89]. The Leading Edge is the first region to make contralateral contact, and matures into the Cadherin-based dorsal midline cell junction of the heart tube (Figure 1C’). Concurrently, the CB soma curves towards the midline to form the ventral seam of the heart tube (reviewed in [72,98]). During this process, the pre-luminal domain is constrained within the heart tube (Figure 1C”). The cardiac cells, cardiomyocytes, enclose the heart lumen. These are flanked by non-contractile pericardial cells (PCs) that function as detoxifying nephrocytes [99,100]. The heart tube is suspended from the epidermis by seven pairs of alary muscles, performing the role of a diaphragm [32,101].

### 4.2. Embryonic and Larval Heart

The embryonic and larval dorsal vessel exhibits anterior-posterior polarisation, and is bisected by intercardiac valve cells to form two distinct regions; the posterior heart, and the narrower anterior aorta (reviewed in [32,94]). The entire heart is comprised initially of 104 CBs, which differentiate into contractile cardiomyocytes, ostial (inlet) cells, and intercardiac valve cells [3,52]. Despite lengthening nearly five-fold by the time of adulthood, the *Drosophila* cardiac system contains no stem cells; there is no CB proliferation or migration post-embryogenesis, nor is there replacement in response to tissue loss or damage [1,11,102].

The embryonic dorsal vessel extends from segment T2 (Thoracic segment 2) to A8 (Abdominal segment 8), with the contractile heart chamber encompassing segments A5 through A8 [62,98,102]. Heart chamber identity is specified by the Hox gene *abdominal-A* (*abd-A*), whereas *Ultrabithorax* (*Ubx*) specifies the posterior aorta [103]. The dorsal vessel exhibits segmental patterning, with a majority of segments formed from six pairs of cardiomyocytes; the two anterior-most pairs express *seven-up* (*NR2F*) and are destined to become ostial cells, whereas the four larger posterior pairs express *tinman* (*NKX2-5*) and will develop as contractile cells (reviewed in [98]). All of these cells are CB derivatives. Throughout the larval phase (first to third instar), thin and thick myofilaments accrue in a circular pattern within cardiac cells, concomitant with lumen expansion [3]. PCs enlarge and decrease in number through apoptosis [62,94,104].

### 4.3. Adult Heart

During metamorphosis, the dorsal vessel experiences extensive remodelling. Twenty of the original 104 CBs undergo apoptosis, resulting in the loss of all cardiac cells in segments A6 through A8, such that segment A5 becomes the terminal heart chamber [3,62,98]. The diameter of the lumen increases as cardiomyocytes enlarge. Aortal myocytes produce additional myofibrils and become contractile [3]. Further differentiation occurs, resulting in the formation of additional ostial cells (from three pairs in larvae to five in adults) and valve cells (from one intercardial valve to three) [94,102]. Lymph gland-like cells associated with the anterior dorsal vessel migrate and differentiate into myoblastic longitudinal cardiac fibres [105].

## 5. The *Drosophila* Heart ECM

### 5.1. Form and Function

ECM and its receptors play a critical role in *Drosophila* cardiogenesis and morphogenesis through the formation of migration corridor cell anchoring points, and through the regulation of signalling and guidance cues that specify the cardiac luminal domain [106]. In the developed larval and adult heart, the ECM maintains connections between cardiomyocytes and adjacent supporting cells such as alary muscles, restores diastolic heart diameter, and helps synchronise myocyte contraction (reviewed in [107,108]).

The ECM is comprised principally of fibrous proteins (e.g., Collagens, Elastin), integrated glycoproteins (e.g., Fibronectin, Perlecan, Nidogen), and growth factors (reviewed in [10]). Key transmembrane cell-adhesion receptors include Integrins, Syndecans, and Dystroglycan [109,110,111,112,113]. The molecular composition of the ECM changes with function and location (reviewed in [10,114]). For the purpose of this review, we shall focus on the Collagen-IV-dominated basement membrane (basal lamina), but not the interstitial ECM, which assists in positioning the heart.

**Figure 2:**
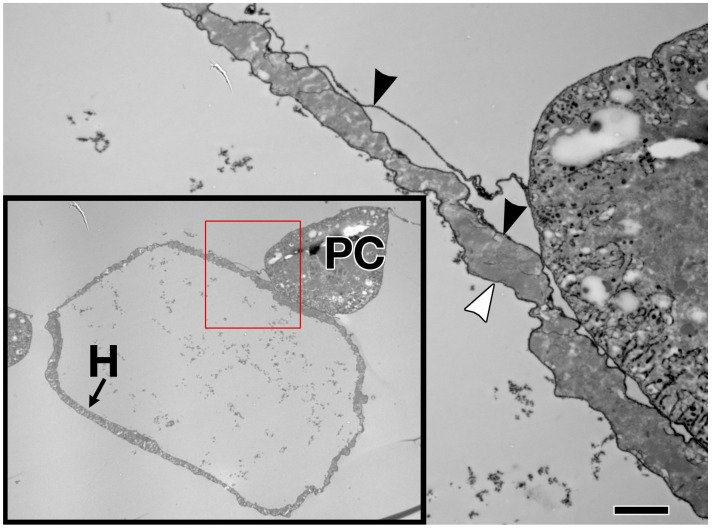
Extracellular matrix in the larval *Drosophila* heart. Enlargement of the cross section shown within the red box (lower left) reveals Ruthenium Red-labelled ECM proteoglycans on both the external (black arrowhead) and luminal (white arrowhead) surfaces of the heart muscle cell, with no endothelium. H: heart muscle; PC: pericardial cell. Scale bar is 2.0 microns.

**Figure 3:**
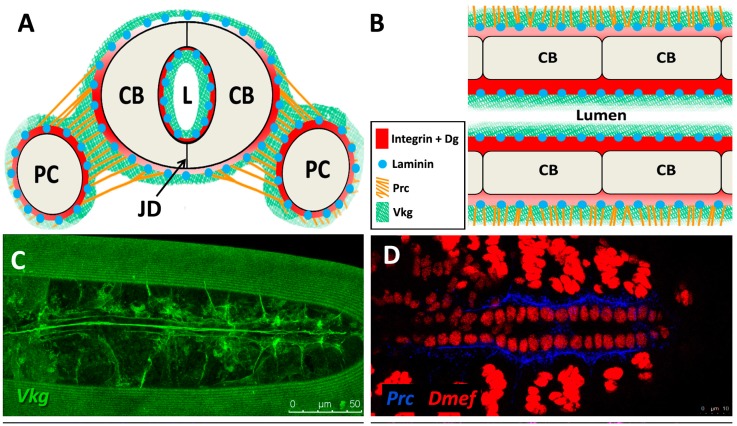

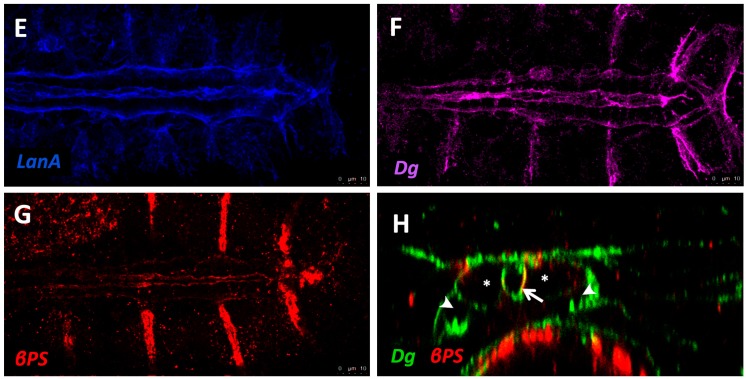
Localisation of ECM proteins and receptors in the embryonic *Drosophila* heart. (**A**) Cross-sectional impression of a stage 17 embryonic dorsal vessel with flanking PCs. The transmembrane receptors βPS-Integrin (βPS) and Dystroglycan (Dg) are present along the cardioblast surface, but are concentrated at the luminal domain. Dg is excluded from the junctional domain. Laminin A (LanA) is present ubiquitously. Viking Collagen-IV (Vkg) localises to the basal lamina and is expressed within the heart lumen. Pericardin (Prc) is absent from the lumen. Dorsal at top. CB: cardioblast; JD: junctional domain; L: lumen; PC: pericardial cell; (**B**) A portion of the embryonic dorsal vessel in coronal view (frontal plane), illustrating CBs but not PCs. Alary muscles have not been included for simplicity; (**C–G**) Distribution of ECM proteins and receptors in stage 17 embryos. Anterior on left. (**C**) Vkg is expressed along the basal lamina of cardiac cells, localised to the luminal and abluminal surfaces. The striated lines running parallel the heart tube is an eggshell artefact; (**D**) Prc forms bundles localised to the abluminal surface of the heart tube, and envelopes the PCs; (**E**) LanA is expressed along the luminal and abluminal cardiac cell surface and within muscle costameres; (**F**) Dg localises to the apical CB surfaces, and to costameres; (**G**) βPS-Integrin is expressed primarily along the CB apical and luminal surfaces, and to a lesser extent along the basal surface; (**H**) Cross-sectional distribution of Dg and βPS-Integrin in a stage 17 embryo. Dg is strongly expressed along the CB luminal surface, and is also present on the abluminal surface, with weak ventral expression. Integrin is strongly expressed along the CB luminal surface, and weakly expressed along the abluminal surface. Dorsal at top. Asterisks label CBs; arrows label the heart lumen; arrowheads label PCs.

**Figure 4:**
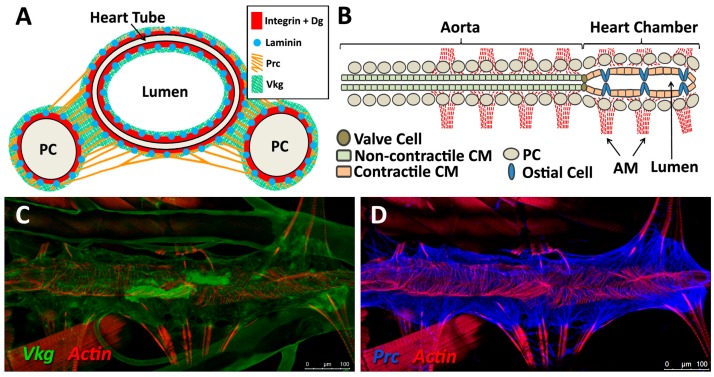

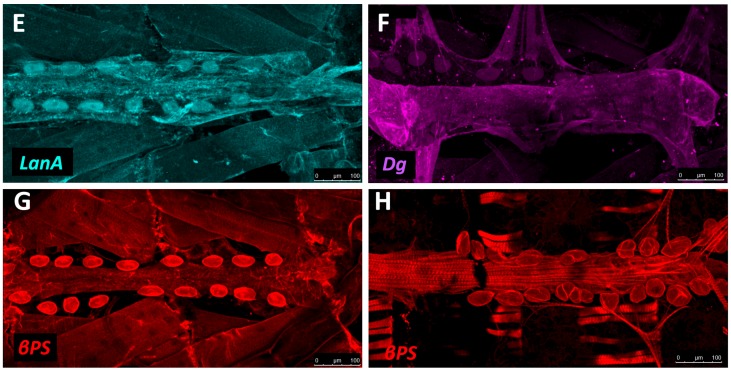
Localisation of ECM proteins and receptors in the larval *Drosophila* heart. (**A**) Cross-sectional impression of a third instar larval dorsal vessel with flanking PCs. The transmembrane receptors βPS-Integrin and Dg are present along the luminal domain and at the abluminal surfaces of the heart tube. LanA is ubiquitous. Vkg localises to the basal lamina and, at lower levels, within the heart lumen. Prc is absent from the lumen. Dorsal at top; (**B**) The larval dorsal vessel in coronal view (frontal plane), illustrating the aorta and the heart chamber, divided by valve cells. The aorta is comprised of non-contractile cardiomyocytes. The heart chamber is wider and formed from contractile cardiomyocytes and non-contractile ostial cells. Anterior on left. AM: alary muscle; CM: cardiomyocyte; (**C–G**) Distribution of ECM proteins and receptors in late third instar larvae. Anterior on left; (**C**) Vkg is expressed along the basal lamina of cardiac cells, as well as in hemocytes and along the alary muscles and trachea. Actin forms parallel myofibrils that envelop the heart tube, and localises to alary muscles; (**D**) Prc networks localise to the abluminal surface of the heart tube, and envelope the PCs; (**E**) LanA is expressed along the luminal and abluminal cardiac cell surface, along the pericardial cell surface, and within muscle costameres (banded pattern); (**F**) Dg is expressed along the heart tube and pericardial cell surface, and within the costameres. Alary muscle tissue is also labelled; (**G**) βPS-Integrin is expressed along the heart tube and pericardial cell surface. Integrin is expressed along costameres (banded pattern); (**H**) Five-day-old adult stained for βPS-Integrin. Integrin is expressed by new longitudinal muscles ventral to the heart tube, and by PC and costameres.

The sheet-like BM forms a compact and ordered proteoglycan matrix abutting endothelial and epithelial cell monolayers (Figure 2). It is dominated by Collagen and Laminin networks, which are further stabilised by Nidogen and Perlecans (reviewed in [10,114]). In most polarised cells, BM proteins are laid down at the basal but not apical surfaces; thus the BM is involved in defining cell apical-basal polarity (reviewed in [115,116]). Migrating cells such as CBs display asymmetric localisation of BM proteins; certain BM constituents and their receptors aggregate to both the basal and luminal domains, and some, such as Multiplexin or Pericardin, are restricted to one domain (Figure 3 and Figure 4) [101,110,117,118,119,120].

### 5.2. Basement Membrane Constituents

Major ECM proteins identified in the *Drosophila* heart and their vertebrate homologues are summarised in Table 1, Table 2 and Table 3. Major components are discussed below.

### 5.3. Structural Proteins

Collagens are the core structural proteins of the ECM, and contribute to the stability and tensile strength of the cardiac tissue (reviewed in [10]). Three conserved genes in *Drosophila* encode BM Collagens; *multiplexin* (*mp*), *Cg25C*, and *viking* (*vkg*), while a fourth, *pericardin* (*prc*), encodes a unique Collagen-like protein (reviewed in [170]). Vkg and Cg25C together form the heterotrimeric *Drosophila* Collagen-IV, which assembles at the basal lamina to provide structural support through linkage with a Laminin-Nidogen complex [119,132]. Prc is a domain-specific Collagen-IV-like cardiac ECM protein required for the adhesion of nephrocytes (PCs) to heart muscle cells [11,101]. Prc is secreted by embryonic PCs and by the larval fat body [101,119]. Prc is recruited to the abluminal domain of cardiac cells by Lonely Heart (Loh), a secreted disintegrin and metalloproteinase with Thrombospondin repeats (ADAMTS)-like protein [11,171]. Multiplexin (Mp) is orthologous with mammalian Collagen-XV/XVIII [172]. It is apically secreted by embryonic cardiac cells, where it localises to the CB luminal domain during the closure of the dorsal vessel [118]. Mp enhances luminal expansion, and its localisation to the posterior dorsal vessel is responsible for the larger heart lumen relative to that of the aorta [118].

Laminin glycoproteins are thought to be the first component of the ECM recruited to the BM [119,128]. They bind the BM cell-surface receptors Integrin and Dystroglycan (Dg), and are necessary for embryonic cell adhesion, migration, and differentiation [128,173,174]. Two heterotrimers are produced in invertebrates compared to 15 or more in mammals [119,175].

Matricellular proteins such as secreted protein acidic and rich in cysteine (SPARC), Thrombospondins (TSPs), and Loh are matrix components serving to link cell surface receptors, proteases, and structural proteins (reviewed in [5,171]). SPARC is an evolutionarily conserved glycoprotein involved in tissue remodelling through the modulation of Collagen deposition and fusion [5,133]. SPARC is secreted by hemocytes to form the basal lamina, where it is implicated in both angiogenesis and angiostasis [132]. TSPs are multi-domain glycoproteins that bind numerous ECM proteins, including Collagens, as well as cell surface receptors, such as Integrins [136,176]. Their inhibitory effect on proteases contributes to matrix stabilisation (reviewed in [5]).

### 5.4. Receptors

Integrins are transmembrane receptors for Collagens and Laminins. They are essential for the formation of cell-matrix linkages, and serve to connect the ECM to the Actin cytoskeleton (reviewed in [109,177]). Integrin signalling is moderated by numerous intercellular linkers, including focal adhesion kinase (Fak), Integrin-linked kinase (Ilk), and Talin [1,141]. During early cardiogenesis, Integrins are necessary to establish the apical ECM, and for the assembly of Collagen, Laminin, and Dystroglycan to form the luminal ECM [110]. The luminal ECM assembles while the CBs are migrating medially (Figure 1B”). Initially, the distribution of Integrin, Laminin, and Collagen is diffuse along the apical and basal CB surface. During the last hours of migration, the activity of the apical Cadherin-rich Leading Edge process increases, and the adjacent apical pre-luminal domain accumulates higher levels of Integrin [89,110]. Apical domain definition and the later emergence of the Leading Edge require Integrin function, as well as that of its cytoskeletal linker protein, Talin [90,110].

Integrin targeting and turnover at the apical ECM is required for the later targeting and retention of the morphogen Slit and its receptor, Robo, to the luminal domain [106,154]. Robo signalling destabilises Cadherin adhesions and acts to delineate the junctional and luminal domains of the heart tube [106]. Loss of function of any of the luminal receptors and ECM components results in the expansion of the Cadherin domain over the apical surface, and a reduced or absent lumen. Other key apical surface receptors are dependent upon Integrin for targeting and stabilisation [110]. These receptors further stabilise and define cardial cell luminal and junctional domains. Syndecan (Sdc), a transmembrane heparin sulfate proteoglycan (HSPG), binds the guidance cue Slit and acts to apicalise the Slit/Roundabout (Robo) complex to promote localised ECM assembly [113,150,152,154]. The Netrin receptors Uncoordinated-5 (Unc5) and Frazzled (homologous with Deleted in Colorectal Cancer) play complementary roles in the regulation of lumen size [157].

Shortly before hatching, the CBs differentiate into cardiomyocytes and re-organise their ECM. Levels of Slit and Robo decline and the Integrin adhesion complex relocates to cardiomyocyte adhesions and muscle costameres. This process is poorly understood, since the development of the cuticle renders the heart largely inaccessible at this stage. During post-embryonic heart growth and morphogenesis, continuous Integrin turnover is necessary for the remodelling of cardiac muscle insertions assoicated with growth and metamorphosis [1].

The Dystrophin-glycoprotein complex (DGC) is involved in signalling and stabilisation of the ECM (reviewed in [178,179]). Dystrophin (Dys) links transmembrane Dg to Actin, connecting the ECM to the cytoskeleton [35,180]. Dg serves as a transmembrane receptor for Laminin [5,115,181,182]. Dg localises to the CB basal and luminal surfaces where it stabilises apical-basal polarity [90,112,183].

## 6. ECM Regulation and Turnover

### 6.1. MMPs and TIMPs

The chemical and biophysical properties of tissues change over time to reflect alterations in cell function, physiology, tension, elasticity, and shape. Constant turnover of the ECM allows for the renewal of proteins, the introduction and post-translational modification of different proteins or carbohydrates, and the remodelling of the density and geometry of protein cross-linking (reviewed in [9]). Integral to this process is the regulated cleavage of ECM proteins by a family of conserved zinc-dependent endopeptidases termed matrix metalloproteinases (MMPs) (reviewed in [42,184]). MMP expression is altered in mammalian heart diseases and during myocardial infarction [185,186,187,188,189,190]. MMP function in the *Drosophila* heart is under study, revealing important roles in lumen establishment and in defining compartment-specific ECM composition [159].

MMPs fall broadly into two classes, transmembrane MMPs and secreted MMP; however the majority of vertebrate MMPs are secreted (reviewed in [41]). MMPs are synthesised as latent zymogens, and are activated by proteolysis or conformational changes via a cysteine-switch mechanism [191]. They are regulated at the post-translational level by endogenous tissue inhibitors of matrix metalloproteinases (TIMPs) (reviewed in [192]). Most vertebrates and invertebrates express multiple MMPs with partially overlapping substrates, which, taken together, are capable of cleaving nearly every kind of ECM protein (reviewed in [184,193]). MMPs are also capable of cleaving non-ECM proteins, including cytokines, chemokines, clotting factors, pericellular proteins, and cell-surface receptors, and can activate other proteinases [40,41,194,195,196]. This substrate versatility sees MMPs involved in myriad developmental and homeostatic processes such as angiogenesis, coagulation and wound healing, Collective Cell Migration, and bone modelling, as well as pathologies such as cancer and heart disease [89,193,194].

### 6.2. ECM Remodelling in Vertebrates

Numerous MMP and TIMP variants are encoded by vertebrates; for instance, humans possess 23 MMPs and four TIMPs, which collectively inhibit all MMPs (reviewed in [40]). Vertebrate MMPs fall into six categories on the basis of substrate affinity or localisation; collagenases, gelatinases, matrilysins, stromelysins, membrane-type (MT)-MMPs, and other MMPs (reviewed in [184]). Collagenase1/2/3 (MMP1/8/13, respectively), secreted GelatinaseA/B (MMP2/9, respectively), Stromalysin1 (MMP3), and Matrilysin1 (MMP7) target substrates within the vertebrate myocardium (reviewed in [169]). All four TIMPS (TIMP1/2/3/4) are expressed within the murine myocardium, with TIMP4 being largely restricted to the heart [197,198,199].

MMPs therefore exhibit some degree of redundancy, and null mutants for a single MMP do not always reveal prominent embryonic phenotypes. In mice, many MMPs appear dispensable for embryogenesis (reviewed in [40]). MMPs are nonetheless required for vertebrate heart development through their role as agents of ECM remodelling and turnover, and careful regulation of MMPs is necessary to maintain cardiac homeostasis post-embryogenesis [161,200,201].

### 6.3. ECM Remodelling in Drosophila

*Drosophila* encodes only two MMPs; a secreted MMP (MMP1), and the trans-membrane MMP (MMP2), both of which are regulated by a singular TIMP [2,202,203,204]. Phylogenetic analysis indicates that the *Drosophila* MMPs are each more similar to their vertebrate homologues than they are to one another [2]. However, MMP1 and MMP2 have partially overlapping substrates; for instance, both are capable of hydrolysing Fibronectin and non-fibrillar Collagens, though MMP1 is incapable of cleaving Laminin [203]. Temporal expression patterns also differ; MMP1 is expressed throughout embryogenesis and larval growth, but not adulthood, whereas MMP2 is expressed from late embryogenesis through adulthood [2,203,204].

In *Drosophila*, MMPs are dispensable for embryonic survival but are critical for tissue remodelling at later developmental stages; embryonic expression of one or both MMPs is required for axonal fasciculation [205], cardiogenesis [159], and tracheal expansion [2].

MMP1 and MMP2 are essential for cardiac development, operating co-operatively to enhance Leading Edge motility during embryonic CB precursor migration, and then in a complementary manner to establish lumen expansion [159]. MMP1 localises to the (pre)luminal domain and constrains the size of the presumptive midline adhesive (junctional) domain. MMP2 localises to the Leading Edge, where it facilitates Collective Cell Migration and CB polarisation by regulating the apical domain of the ECM. MMP2 is required to restrict lumen-specific ECM proteins (e.g., Slit, Dystroglycan, and Collagen) to the luminal domain.

In both *Drosophila* and vertebrates, MMPs play analogous roles in heart morphogenesis and cardiomyopathy. Future studies on this and other genetic models should reveal more about how cardiac ECM remodelling contributes to stress and aging, and identify therapeutic targets for diagnosis or therapy.

## 7. Cardiac Aging and ECM Disruption

Cardiac performance declines progressively in aging *Drosophila* and vertebrates (reviewed in [35,206]). Mis-regulation of ECM components in the form of altered expression or accumulation is characteristic of cardiac aging and dysfunction. Changes in the synthesis, deposition, and degradation of cardiac ECM are correlated with fibrosis and hypertrophy that mark the aging or diseased vertebrate heart (reviewed in [5,207,208]). Fibrosis results from an accretion of ECM proteins (loss of homeostasis) following accumulation and differentiation of cardiac fibroblasts into pro-fibrotic myofibroblasts (largely responsible for fibrillar Collagen synthesis [209]) that renders the heart less elastic, and is correlated with reduced or non-heterogeneous electrical conduction [210,211], elevated incidence of arrhythmia [212,213], and heart failure [214,215]. Hypertrophy emerges in the backdrop of progressive myocyte loss and vascular stiffening in aging vertebrates [216]. Cardiac dilation similarly arises from alterations in ECM regulation and involves a weakening of Collagen-cytoskeletal linkages, though the resultant increase in luminal volume occurs without a compensatory increase in chamber wall thickness, as is the case in hypertrophy, resulting in impaired muscle integrity throughout development and growth [217]. Dilation is correlated with interstitial fibrosis [7,218] and reduced contractility that may result in diminished cardiac output and decreased life expectancy [1,4]. These phenomena impair myocardial compliance and contribute to contractile dysfunction, impeding relaxation and increasing diastolic pressure (for a detailed review, see [206,215,219]).

Unfortunately, many mutations of structural ECM genes prove embryonic-lethal for both vertebrates and *Drosophila*, hampering the examination of adult phenotypes. However, temporal activation of gene expression in *Drosophila* (e.g., by employing the temperature-sensitive Gal80^TS^ inhibitor of Gal4) can circumvent this constraint.

### 7.1. Altered Expression or Deposition of Structural Proteins

Collagens accumulate naturally in the aging vertebrate heart [108,220,221], and changes in Collagen levels are noted during heart failure [222]. Concomitant over-expression of myocardial Collagen-VI α2 and the guidance receptor Down syndrome cell adhesion molecule (Dscam) results in cardiac defects in both flies and mammals; adult *Drosophila* show a reduction in heart rate and arrhythmic contractions due to altered cell-substrate adhesion, and mice develop cardiac hypertrophy and atrial-septal defects [146]. *Drosophila* with reduced Mp (Collagen-XV/XVIII) exhibit embryonic luminal defects and impaired contractility (reduced fractional shortening) as adults [118], while mice deficient in Collagen-XV develop cardiomyopathies and exhibit myofibrillar disorganisation and cardiac stiffening [125]. *Drosophila* heterozygous for *Cg25C* (Collagen-IV α1) manifest sarcomeric defects along the musculature of the adult oviduct, and embryos have abnormal Perlecan localisation along the cardiac BM and accumulation within hemocytes [119]. A reduction of either Collagen-IV (Vkg or Prc) or Laminin expression within PCs and cardiomyocytes with RNA interference mitigates age-related defects; the thickness of the cardiac BM is reduced and lifespan increases [121]. Compared to control adults, individuals with reduced Collagen-IV or Laminin experience a less severe reduction in systolic and diastolic diameter with age, and show increased fractional shortening.

Loh (ADAMTSL-6) and Prc (Collagen-IV-like protein) mutants reveal a different class of cardiac phenotypes, including PC detachment from the heart tube, compromised circulation, and reduced lifespan [11]. *Drosophila* reared on a high sucrose diet accumulate Prc within cardiac tissues and show myofibrillar disorganisation; these develop cardiac arrhythmia, asystoli, and fibrillation as adults, and exhibit impaired contractility similar to the dilated cardiomyopathy observed in diabetic mammals [223].

### 7.2. Mis-Regulation of Receptors and Linker Proteins

Integrin levels increase in the aging *Drosophila* heart [141] and mammalian vascular smooth muscle [142], but are reduced in older murine hearts [220]. In *Drosophila*, a mild reduction of β1-Integrin or Integrin-linked kinase (Ilk) expression in cardiomyocytes appears cardioprotective and may mitigate the effects of age-dependent Integrin accumulation, resulting in reduced incidence of arrhythmia with age, as well as decreased myocardial stiffness and increased lifespan. In rat cardiac fibroblasts, reduction of Ilk is correlated with a decline in the markers of cellular senescence [224]. However, the β1-Integrin/Ilk complex is necessary for normal cardiac development and contractile activity, and inhibition within cardiomyocytes and PCs beyond a certain threshold results in cardioblast adhesion defects and arrhythmia in *Drosophila* [141], while β1-Integrin/Ilk knock-out mice develop myocardial fibrosis, cardiomyopathy, and heart failure [143,225]. Over-expression of β1-Integrin within young adult *Drosophila* hearts increases myocardial stiffness and accelerates heart aging, leading to arrhythmia, decreased diastolic diameter, and decreased fractional shortening common in older hearts [141]. Though cardiac stiffening increases with age and is correlated with failing hearts, inelasticity in and of itself is not necessarily indicative of dysfunction [226]. For instance, up-regulation of the focal adhesion protein Vinculin reinforces the *Drosophila* cardiac cytoskeleton, resulting in reduced cardiac diameter but increased shortening and lengthening velocities, thus mitigating age-related decline in contractility and increasing longevity [227]. Careful regulation of ECM proteins is therefore necessary to ensure cardiac health.

Inhibition of Dystrophin (Dys) expression in *Drosophila* results in cardiac dilation and reduced fractional shortening, reminiscent of dilated cardiomyopathy in mammals [71]. Individuals further experience a more rapid onset of age-dependent myofibrillar disorganisation and decreased lifespan. In mammals, mutation of the *dys* gene is a cause of muscular dystrophy (MD), in which cardiomyopathies and heart failure are symptomatic, and cardiac dilation and arrhythmia are observed (reviewed in [228]).

**Figure 5:**
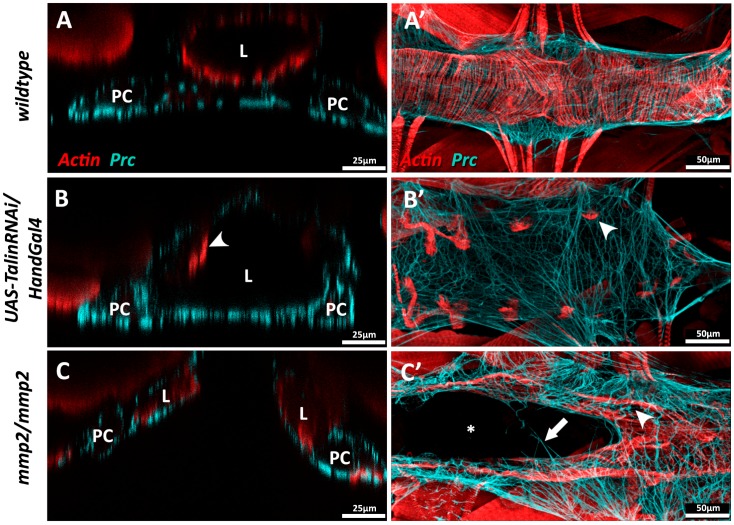
Mis-regulation of ECM proteins in *Drosophila* results in cardiac dilation or cardia bifida. (**A–A’**) Wildtype third instar dorsal vessel in cross-sectional (**A**) and coronal view (**A’**). Actin (red) forms regular transverse myofibrils along the length of the heart tube. Abluminal Prc networks (cyan) envelop the heart tube and pericardial cells (PCs); (**B–B’**) Dorsal vessel of a third instar *UAS-TalinRNAi/HandGal4* larva in which Talin expression has been inhibited via RNA interference in cardiomyocytes and PCs during late embryogenesis and throughout larval growth. (**B**) Cross-sectional view illustrating dilation of the cardiac lumen. Actin myofibrils do not fully envelop the heart tube, and appear constrained to the lateral regions of the vessel (arrowhead) (**B’**) Actin myofibrils are fewer in number and reduced in length (arrowhead), and Prc bundles are more broadly distributed; (**C–C’**) Homozygous third instar *mmp2^w307^*phenotypic null mutants show cardia bifida. (**C**) Cross-sectional view reveals two discrete heart vessels. The Prc network envelops the abluminal domains of both vessels. (**C’**) Portions of the *mmp2* mutant heart chamber are split, while other regions show successful contralateral contact between cardioblasts to enclose a singular lumen. A small number of Prc bundles connect the two discrete heart vessels (arrow). The Actin cytoskeleton appears disorganised; myofibrils envelop the heart vessels but gaps are visible between parallel fibres (arrowhead). L: heart lumen; PC: pericardial cell.

*Drosophila*, unlike vertebrates, possesses but a single Talin gene (*rhea*) [47,229]. Ubiquitous loss of Talin function during *Drosophila* embryogensis compromises cardioblast polarity and promotes the formation of ectopic cardiac lumens [90], while localised inhibition of Talin within the myocardium causes loss of contact between neighbouring myocytes, as well as accumulation of Prc (Collagen-IV) and reduced longevity [1]. Myocardial Talin reduction results in compromised contractility (reduced cardiac output) and cardiac dilation that resembles dilated cardiomyopathy in vertebrates (Figure 5B).

### 7.3. Mis-Expression of MMPs

Both *Drosophila* MMPs (MMP1 and MMP2) are required for normal cardiac development. In embryonic MMP1 mutants, the junctional domain of the *Drosophila* cardioblast (CB) expands at the expense of the luminal domain; these individuals exhibit less organised cardiomyocyte migration and possess a small heart lumen [159]. In MMP2 mutants, luminal proteins are broadly expressed along the entire surface of the CB, and no junctional domain is formed. MMP2 mutants that survive embryogenesis may display luminal defects such as cardia bifida, wherein portions of the heart are split into bilateral tubes (Figure 5C) [159,160].

MMP expression is altered in aged and diseased vertebrate hearts. In older humans, for example, levels of myocardial MMP2/7 and TIMP1/2/4 (from circulating plasma) are increased, whereas those of MMP9 are lowered, resulting in the net reduction of cardiac MMP expression relative to that of TIMP, representing a diminished remodelling capability [230]. In rats and mice experiencing myocardial fibrosis during systolic heart failure or dilated cardiomyopathy, MMP1/2/3/7/8/9/12/13/14 and TIMP1/2 mRNA expression is shown to increase, while TIMP3/4 protein levels drop (reviewed in [4]). Post-infarction cardiac remodelling in vertebrates is dictated in large part by the activities of specific MMPs. During heart failure, MMPs act to promote tissue remodelling and so increase susceptibility to fibrillation [231,232]. For example, MMP2/9, which promote the migration of cardiac progenitor cells during embryogenesis, are up-regulated in the adult pig left ventricle after myocardial infarction [164,201]. Healthy canine hearts constitutively express TIMP3/4 mRNAs, but not MMP mRNAs or those of TIMP1/2 [161]. However, in those manifesting abnormalities, MMP1/2/3/9/13 and TIMP1/2/3/4 mRNAs are up-regulated in myocardial tissue. Moreover, diverse cardiac disorders in canines reveal non-identical mRNA expression profiles for these MMPs and TIMPs within different cardiac tissues, suggesting that specific MMPs and TIMPs play specific roles in certain diseases [161].

## 8. Conclusions

Cardiac disorders are prevalent in many species of veterinary significance. Since the remodelling of ECM is a common thread between cardiac disorders and diseases, the importance of elucidating the mechanism of ECM regulation cannot be understated. The homology of developmental pathways and molecular mechanisms, relative simplicity, short generation time, and availability of genetic tools make *Drosophila* an indispensible model for the study of cardiogenesis and heart remodelling. A number of vertebrate disorders, such as cardiac dilation and ECM weakness, have complementary morphology in *Drosophila*, and are triggered by the loss or over-expression of the same genes. The studies reviewed here reveal that several ECM proteins and receptors, such as Collagens and the Integrin adhesion proteome, are required continuously to permit the heart to form, grow, and adapt to changes in activity or load. Other ECM proteins reveal their function with age, when reduced elasticity correlates with reduced lifespan. Further understanding of the physiological and biomechanical response to disorders of the ECM in *Drosophila* may reveal future insights into mammalian cardiovascular diseases.

### Note Added in Proof

Since submission of this manuscript, it has been demonstrated that both *Drosophila* MMPs (MMP1 and MMP2) can be secreted or remain membrane-bound (LaFever, K. S.; Wang, X.; Page-McCaw, P.; Bhave, G.; Page-McCaw, A. Both Drosophila matrix metalloproteinases have released and membrane-tethered forms but have different substrates. *Sci. Rep.* **2017**, 7, 44560) [233].

## Figures and Tables

**Table 1:** Structural proteins of the BM.

Drosophila Protein	Vertebrate Homologue	Functional Role	Drosophila Heart Phenotype	Vertebrate Heart Phenotype
**Collagen-IV**	Collagen-IV	Cell adhesion, Basal Lamina	Mutants exhibit alary muscle and PC detachment, and accumulation of Perlecan within hemocytes [119]. RNAi knock-down in the heart mitigates age-related decline in fractional shortening and increases longevity [121].	Up-regulated post-infarction during repair in rats [122]. Not restricted to the BM during DCM in humans or myocardial infarction in rats; found in fibrotic lesions [123,124].
**Prc**	N/A (Collagen-IV α-like)	Cell adhesion	Mutants exhibit cardiomyocytes that do not properly polarise and fail to align, detachment of PCs and alary muscles from the heart tube, and reduced lifespan [11,101]. RNAi knock-down in the heart mitigates age-related decline in fractional shortening and increases longevity [121].	N/A (see Collagen-IV)
**Mp**	Collagen-XV/XVIII	Lumen expansion	Loss of function results in a small lumen and diminished fractional shortening; over-expression results in increased lumen size or development of ectopic lumens [118].	Collagen-XV deficiency in mice causes disorganised fibrillar Collagen, increased left ventricle (LV) myocardial stiffness, cardiac hypotrophy, aberrant cardiomyocyte structure, and cardiomyopathy [125]. Collagen-XVIII deficiency in rats results in extended cardiac BM, adverse remodelling, and heart failure post-infarction [126,127]. Up-regulated after hypoxia [127].
**Laminin**	Laminin	Cell adhesion, Collagen assembly	Mutants show failed accumulation of Perlecan, Collagen-IV, and Prc [128], gaps between CBs, breaks in the cardiac tube, a small lumen, muscle attachment defects [119], and dissociation of PCs from heart tube [129]. RNAi knock-down in the heart mitigates age-related decline in fractional shortening and increases longevity [121].	Decreased expression during ischemic heart failure [130]. Mutant mice develop cardiomyopathy and cardiac hypertrophy [131].
**SPARC**	SPARC	Collagen assembly	Knock-down causes disorganisation of Laminin, failed Collagen-IV assembly, and reduced heart contractility [132,133].	Up-regulated in older mice, causing ventricular stiffness [134]. Up-regulated in infarcted mouse and canine hearts (protective role) [5,14].
**TSP**	TSP-3/4	Cell adhesion	Mutants exhibit detachment of muscle cells from tendons [135,136].	Canine TSP-1 is up-regulated post-infarction [137]. TSP-3/4 up-regulated during remodelling and pressure overload [5,138]. Mice mutant for TSP-1 exhibit increased fibrosis, and detrimental LV remodelling post-infarction [137]. Mice mutant for TSP-2 are more prone to cardiac rupture post-infarction and exhibit DCM and fibrosis with aging [5]. Mice mutant for TSP-4 deposit more ECM , leading to fibrosis and decreased contractility [139].
**Loh**	ADAMTSL6	Cell adhesion	In mutants, Prc does not localise between PCs and heart tube; PCs and alary muscles detach from the heart tube, and lifespan is reduced [11].	Over-expression results in the accumulation of Fibrillin-1-containing ECM microfibrils in mice [140].

**Table 2:** Receptors and signalling cues of the BM.

Drosophila Protein	Vertebrate Homologue	Functional Role	Drosophila Heart Phenotype	Vertebrate Heart Phenotype
**β-Integrin**	β-Integrin	Cell-ECM linker, adhesion signalling	Age-dependent up-regulation results in increased myocardial stiffness [141]. Mutants exhibit reduced CB Leading Edge activity, mis-aligned CBs, failed localisation of βPS-Integrin to CB surface, ectopic Prc and Slit, and lack luminal domains [110].	Age-dependent up-regulation results in increased aortic stiffness in monkeys [142]. Knock-down mice causes fibrosis, reduced LV contractility, and DCM [143].
**Dg**	Dg	Cell-ECM linker	Mutants exhibit mis-expression of epithelial cell apical markers in basal domain and loss of anterior-posterior polarity, as well as age-related muscular degeneration [112,144].	In humans, reduced Dg glycosylation weakens ECM attachment, and causes DCM [145].
**Dscam**	Dscam	Cell adhesion and signalling	Mutants exhibit reduced CB Leading Edge velocity, disrupted (non-continuous) heart lumen [89]. Concomitant over-expression with Collagen-VI reduces heart rate and causes arhythmia/asystole [146]. The large number of DSCAM isoforms constrains extension by homology to vertebrates [147,148].	Candidate Down syndrome congenital heart defect (CHD) gene in humans [149]. Mouse over-expression causes atrial-septal defects and LV thickening (CHD, hypertrophic cardiomyopathy) [146].
**Sdc**	Sdc	Cell adhesion and signalling	Mutants and RNAi knock-downs exhibit gaps between CBs and between PCs, mis-localisation of Prc, and failure of CBs to polarise (no apicalisation of Robo/Slit) [150].	Contributes to post-infarction fibrosis ECM stiffness and cardiac hypertrophy (reviewed in [151]).
**Robo**	Robo	Morphogen receptor	Mutants exhibit gaps between CBs at the midline, and small, intermittent, or no lumen [106,152,153], and reduced CB migration velocity [154].	Mutant mice exhibit cardiac valve and septum morphogenesis, ectopic pericardial cavities, and caval vein malformation [155,156].
**Slit**	Slit	Secreted morphogen	Mutants exhibit CB mis-alignment and gaps between CBs at the midline, resulting in small, intermittent, or no lumen [152,153], as well as cardiac tube lesions and reduced CB migration velocity [154]. Over-expression causes the formation of ectopic lumens [106].	Mutant mice exhibit reduced angiogenesis, cardiac valve and septum morphogenesis, ectopic pericardial cavities, and caval vein malformation [155,156].
**Fra**	DCC	Morphogen receptor	Mutants exhibit CB mis-alignment at the midline, and defective contralateral attachments between CBs results in an open or enlarged lumen, or no lumen [157].	Cardioprotective role in elevating nitric oxide production [158].

**Table 3:** Remodelling proteases of the BM.

Drosophila Protein	Vertebrate Homologue	Functional Role	Drosophila Heart Phenotype	Vertebrate Heart Phenotype
**MMP1/2**	MMP1-28	ECM protease	MMP1 mutants have a reduced heart lumen, and embryonic CB migration is less organised; in MMP2 mutants embryonic CBs do not form cell junctions, resulting in reduced or absent heart lumen; surviving larvae show cardia bifida [159,160].	MMP1/2/3/7/8/9/14 show increased protein expression in DCM (reviewed in [4]). MMP1/2/3/9/13 are up-regulated in canines exhibiting cardiac abnormalities [161]. MMP2/9 are up-regulated in infarcted mouse and pig hearts, with depletion resulting in decreased odds of cardiac rupture [162,163,164].
**TIMP**	TIMP1-4	Inhibitor of ECM proteases	Ectopic ectodermal expression of TIMP inhibits heart lumen formation [159].	Mouse TIMP mutants experience adverse remodelling / increased ECM degradation post-infarction [165,166]; TIMP1 mutants develop cardiac hypertrophy [167] and TIMP3 mutants develop DCM [168]. TIMP1/2/3/4 are up-regulated in canines exhibiting cardiac abnormalities [169].
